# Hollow Bio-derived Polymer Nanospheres with Ordered Mesopores for Sodium-Ion Battery

**DOI:** 10.1007/s40820-020-0370-1

**Published:** 2020-01-21

**Authors:** Yan Ai, Yuxiu You, Facai Wei, Xiaolin Jiang, Zhuolei Han, Jing Cui, Hao Luo, Yucen Li, Zhixin Xu, Shunqi Xu, Jun Yang, Qinye Bao, Chengbin Jing, Jianwei Fu, Jiangong Cheng, Shaohua Liu

**Affiliations:** 1 State Key Laboratory of Precision Spectroscopy and Department of Materials, School of Physics and Electronic Science, East China Normal University, Shanghai, 200241 People’s Republic of China; 2 Department of Physics and Astronomy, Shanghai Jiao Tong University, Shanghai, 200240 People’s Republic of China; 3 School of Chemistry and Chemical Engineering, Shanghai Jiao Tong University, Shanghai, 200240 People’s Republic of China; 4 Center for Advancing Electronics Dresden (cfaed) and Department of Chemistry and Food Chemistry, Technische Universität Dresden, 01062 Dresden, Germany; 5 School of Materials Science and Engineering, Zhengzhou University, Zhengzhou, 450052 People’s Republic of China; 6 State Key Lab of Transducer Technology, Shanghai Institute of Microsystem and Information Technology, Chinese Academy of Sciences, Shanghai, 200050 People’s Republic of China

**Keywords:** Self-assembly, Biomimetic synthesis, Mesoporous polymer, Ferric phytate, Sodium-ion battery

## Abstract

Bio-inspired hierarchical self-assembly provides elegant and powerful bottom-up strategies for the creation of complex materials. However, the current self-assembly approaches for natural bio-compounds often result in materials with limited diversity and complexity in architecture as well as microstructure. Here, we develop a novel coordination polymerization-driven hierarchical assembly of micelle strategy, using phytic acid-based natural compounds as an example, for the spatially controlled fabrication of metal coordination bio-derived polymers. The resultant ferric phytate polymer nanospheres feature hollow architecture, ordered meso-channels of ~ 12 nm, high surface area of 401 m^2^ g^−1^, and large pore volume of 0.53 cm^3^ g^−1^. As an advanced anode material, this bio-derivative polymer delivers a remarkable reversible capacity of 540 mAh g^−1^ at 50 mA g^−1^, good rate capability, and cycling stability for sodium-ion batteries. This study holds great potential of the design of new complex bio-materials with supramolecular chemistry.

**Electronic supplementary material:**

The online version of this article (10.1007/s40820-020-0370-1) contains supplementary material, which is available to authorized users.

## Highlights

- A novel coordination polymerization-driven hierarchical assembly approach for spatially controlled fabrication of phytic acid-based bio-derivatives was developed.
- The resultant ferric phytate bio-derived polymer featured hollow nanosphere architecture, ordered meso-channels, high surface area, and large pore volume, as anode material, delivering a remarkable electrochemical performance.

**Electronic supplementary material:**

The online version of this article (10.1007/s40820-020-0370-1) contains supplementary material, which is available to authorized users.

## Graphical abstract

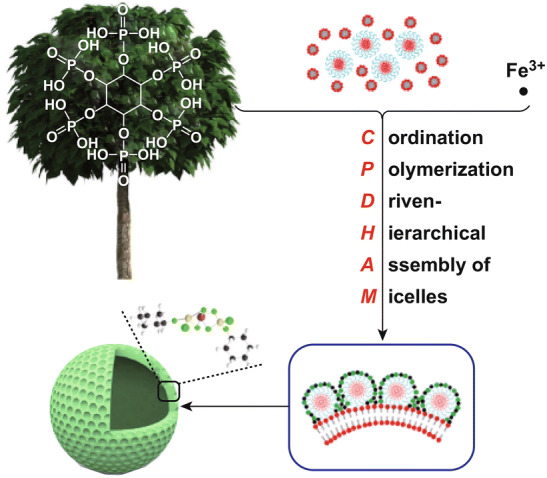

## Introduction

Nature is a powerful designer that creates numerous wonderful works. One of the most striking masterpieces is the hierarchical structure of organism, deriving many unique properties [1, 2]. For instance, the hierarchical structure of butterfly wings can interfere and diffract with sunlight, thus presenting brilliant colors [3]. The three-layered stratified structure furnishes the shells of the *Pinctada margaritifera* with incredible mechanical strength, durability, and toughness [4]. The multi-scale hierarchical pore structure of trees provides channels for fast nutrient transport [5, 6]. Therefore, construction of bio-inspired materials with hierarchical structure is highly desirable for achieving various customized functions. Soft matter with abundant groups and diverse shapes, as a kind of versatile structure directing agents, is capable of manipulating the growth of discrete precursors by assembling and then instruct them to mineralize into final products with complex architecture [7, 8]. However, the build-up of hierarchies needs to ensure the homogeneity of the soft matter assemblies for the next higher levels, in that the polydispersity would be amplified and then lead to a kinetic obstacle for the further hierarchical co-assembly with the increase in size and complexity of the building blocks [9, 10]. As a result, precisely controlled hierarchical assembly of the multiple soft matters different in size and property is still a significant bottleneck.

Recently, because of non-toxicity, easy access, wide availability, and diversity, bio-derived polymers derived from metal ion and bio-resource precursor (such as polyphenol, porphyrin, and polyacid) are emerging as attractive functional materials. In particular, as promising rechargeable electrodes materials, the inherent porous architecture of bio-derived polymer frameworks resulting from organic molecular is beneficial for hosting cations [11–13]. Furthermore, large accessible mesopores would be highly desired for their application in energy storage fields as advanced electrodes materials, because regular mesoscale nanopores would provide for materials with higher specific surface area, more exposed active sites, and specific ion transport channels [14]. Unfortunately, the synchronous control of mesoscale structure and morphology of bio-compounds from natural sources still remains unrealized so far, maybe due to the strong chelation with metal ions, continuous nucleation, and fast growth of precursors [15–17].

Here, we develop a novel coordination polymerization-driven strategy to manipulate the hierarchical assembly of two kinds of amphiphilic soft matters for spatially controlled fabrication of ferric phytate bio-derivatives. The strong coordination among phytic acid and ferric ion promotes the discrete assemblies of pentadecafluorooctanoic acid (PFOA) and polystyrene-*b*-poly (ethylene oxide) (PS-*b*-PEO) into hollow vesicles surrounding with spherical micelles, which further confine the growth of precursors. For the first time, we have successfully achieved the synchronous control of mesoscale structure and morphology for bio-compounds. The resultant ferric phytate polymer nanomaterials are characterized by mesoporous hollow spherical architecture (hereafter termed as mFePA-HS) with a high surface area of 401 m^2^ g^−1^, large pore volume of 0.53 cm^3^ g^−1^, uniform mesoscale channels of ~ 12 nm. In addition, iron-based bio-resourced materials have the advantages of rich resources, environmentally friendly, low cost, good safety, and high theoretical specific capacity, so it can be used as anode or cathode in secondary batteries [18, 19]. As a proof of concept, the bio-derivative was first explored as an advanced anode material, delivering excellent capacity, good rate capability, and cycling performance for sodium-ion batteries. This strategy has opened new avenues for spatially controlled construction of bio-based functional materials.

## Experimental Section

### Chemicals and Materials

BCP of PS_96_-*b*-PEO_114_ was synthesized in our laboratory. Phytic acid solution, Iron (III) *p*-toluenesulfonate, and pentadecafluorooctanoic acid were purchased from Maclin, Aladdin and Ark, respectively. Tetrahydrofuran and ethanol were purchased from Greagent. All chemicals were used without further purification. Deionized water was used for all experiments.

### Synthesis of mFePA-HS

Typically, 0.05 g of PS_96_-*b*-PEO_114_ BCP was dissolved in 1 mL THF. Then, 1 mL H_2_O was added to the above solution by dropwise addition at the rate of 1 min one drop. After continuous stirring for half an hour, 7 mL of H_2_O was poured into the solution thus forming the micelles. After stirring, 0.016 g pentadecafluorooctanoic acid solution (1.1 wt% in ethanol) was added, followed by the addition of 0.040 g iron *p*-toluenesulfonate (11.8 wt% in water) and the solution turned yellow. After stirring for 4 h, the pH of the synthetic medium was increased up to 3 by adding 0.140 mL 1 M ammonia. Finally, the reaction vessel was put into an ice bath and 0.010 mL phytic acid solution (70 wt% in water) was added into it. After continuous stirring, the color of solution slowly faded, indicating the formation of ferric phytate polymer. The pure mFePA-HS was obtained after removing the BCPs, PFOA, and excess ions by repeatedly washing with THF, ethanol, and the resulting product was further dried at 150 °C for 5 h.

### Synthesis of Hollow Ferric Phytate Nanosphere

First, 0.016 g pentadecafluorooctanoic acid solution (1.1 wt% in ethanol) was mixed with 1 mL THF and 8 mL H_2_O under stirring for 30 min, followed by the addition of 0.040 g iron *p*-toluenesulfonate (11.8 wt% in water). Then, the reaction vessel was put into the ice bath and 0.010 mL phytic acid solution (70 wt% in water) was added into it. After continuous stirring, the hollow ferric phytate was obtained after removing the PFOA, by repeatedly washing with ethanol.

### Synthesis of Blank-FePA

First, 0.040 g iron *p*-toluenesulfonate (11.8 wt% in water) was added into a mixed solution of 1 mL THF and 8 mL H_2_O. Then, after continuous stirring for 30 min, 0.010 mL phytic acid solution (70 wt% in water) was added into the reaction vessel. Finally, the pH of synthetic medium was increased up to 3 and the blank ferric phytate was obtained by centrifugation.

### Characterization and Measurements

The morphology and structure of the mFePA-HS were investigated by scanning electron microscopy (SEM, S-4800) and transmission electron microscopy (JEM-2100F). Infrared spectra were recorded on an FTIR Spectrometer (Nicolet iS50 FTIR, Thermo). Powder XRD patterns were recorded on a Bruker X-ray diffractometer (Smartlab SE) equipped with Cu-Ka radiation (40 kV, 20 mA) at a rate of 10° min^−1^ over the range 10–80 (2*θ*). Nitrogen absorption isotherms were measured at 77 K on a Quantachrome 9ASIQMUTV02UT-6. Prior to measurements, all samples were degassed in a vacuum at 120 °C for at least 12 h. Specific surface area was determined by standard Barrett–Emmett–Teller (BET) method in the relative pressure range of 0.05–0.9 *P*/*P*_o_ and pore size distribution was analyzed by density functional theory (DFT). X-ray photoelectron spectroscopy (XPS) measurements were performed in a surface analysis system inducting a sample analysis chamber with the pressure of 3 × 10^−10^ mbar, and the analyzer is Scienta-R3000. The spectra were calibrated by determining to Au 4*f*_7/2_ peak position of the clean Au foil. Thermogravimetric analysis was performed on a TGA/SDTA851e instrument in an air atmosphere. Cryogenic transmission electron microscopy was performed on Tecnai F20 from FEI. Zeta-potential measurements were taken on Malvern Zetasizer Nano ZS.

### Electrochemical Measurements

Electrochemical properties were tested by using a CR2032 coin cell. The working electrodes were prepared by mixing 70 wt% composite materials, 20 wt% carbon black as a conductive agent and 10 wt% polyvinylidene difluoride (PVDF) as binder. After coating the slurry on a Cu foil, the electrodes were dried at 80 °C for 6 h and then transferred to a vacuum oven at 120 °C for 12 h. The loading amount of the electrode was kept at ~ 1.5 mg cm^−2^. The assembly of all coin cells was conducted in an argon-filled glovebox, sodium metal was used as the counter and reference electrode, 1 M NaClO_4_ in the mixture of EC/PC (1:1) was used as electrolyte, and glass microfiber filters from Whatman were used as separator. Electrochemical tests were conducted by a Land battery system; the voltage window was 0.001–3.0 V. Cyclic voltammetry (CV) tests were carried out by using the three-electrode customized cell. The CV measurements were taken on a CHI760E electrochemical workstation at a scan rate from 0.1 to 10 mV s^−1^.

## Results and Discussion

**Fig. 1:**
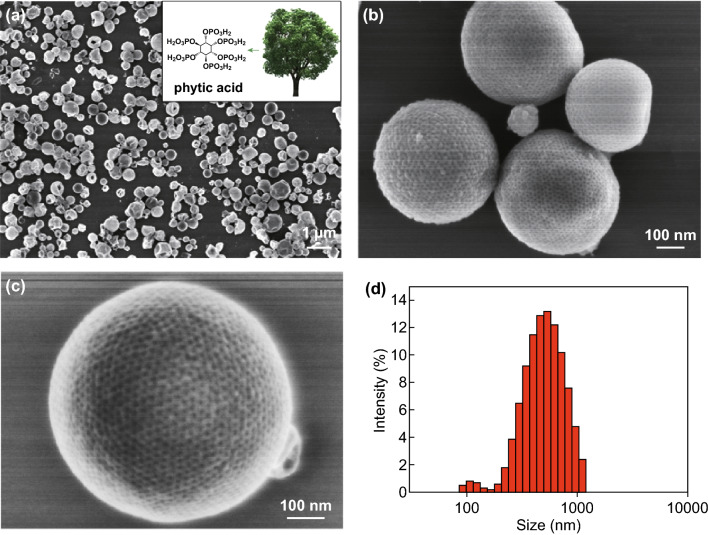
Morphology of mFePA-HS. **a**–**c** SEM images of mFePA-HS at different magnifications. **d** The particle size distribution of mFePA-HS measured by dynamic light scattering

SEM images reveal the well-defined spherical morphology of the resultant ferric phytate polymer nanomaterial. As shown in Fig. 1a–c, the ordered mesopores are uniformly distributed on the surface of the nanosphere, and the inserted picture in Fig. 1a indicates that the phytic acid comes from biology. The pore size and wall thickness of mesopores are ~ 12 and ~ 10 nm, respectively. The size distribution of the particles was measured by dynamic light scattering (DLS), showing the average particle size of ~ 450 nm (Fig. 1d). By contrast, only irregular ferric phytate nanoparticles were obtained at the absence of block co-polymer (BCP) PS-*b*-PEO and PFOA (blank-FePA) under the same conditions.

**Fig. 2:**
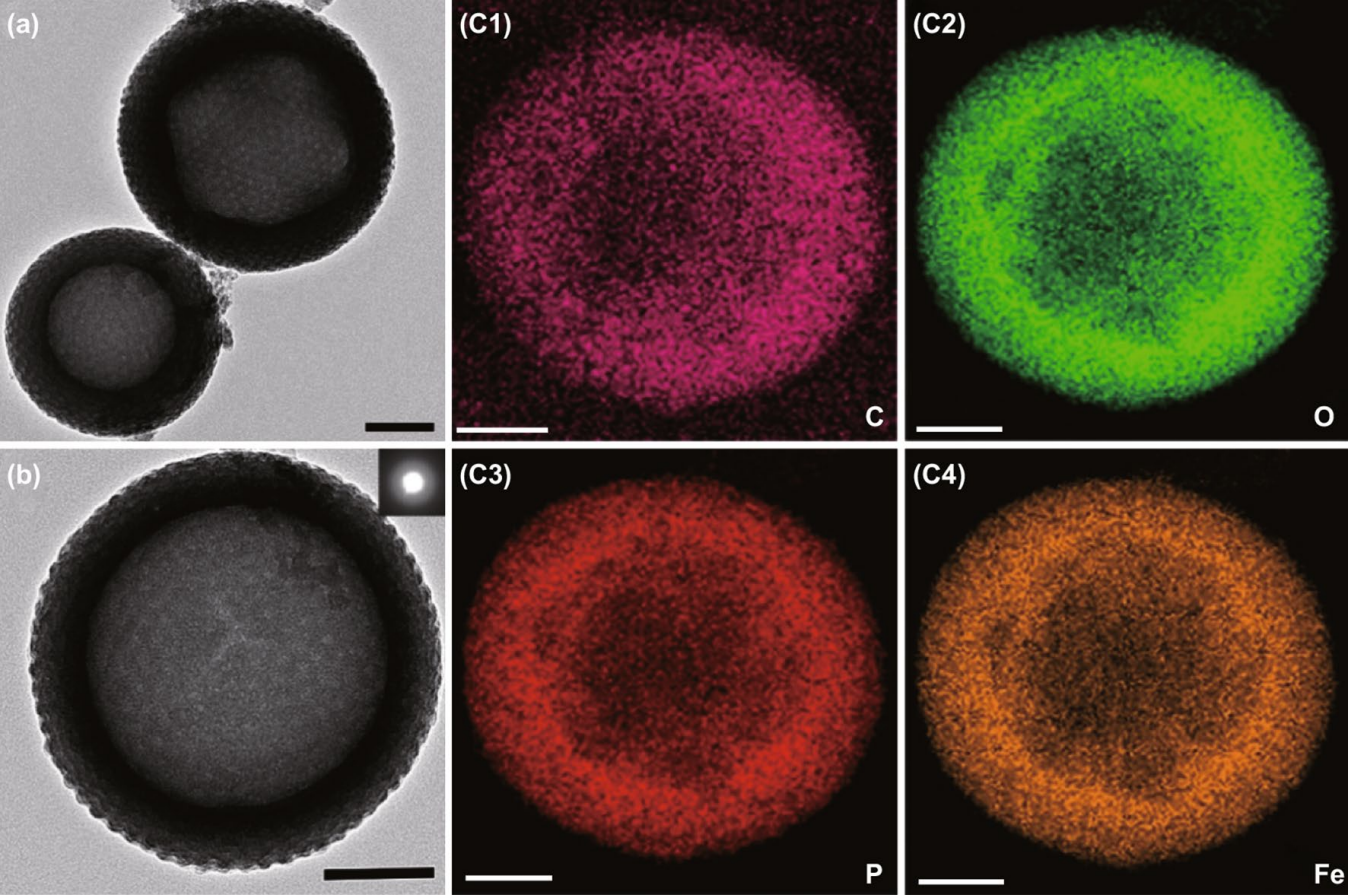
Structure of mFePA-HS. **a**, **b** TEM images. **c** EDX element mappings of mFePA-HS (scale bar 100 nm)

Interestingly, TEM images show the hollow structure of the ferric phytate nanospheres, and the thickness of the shells is approximately from 15 to 55 nm (Fig. S2). Furthermore, the morphology of mFePA-HS varies with the thickness of the shell, which exhibits a regular hollow structure with ordered mesopores when the shell is thick enough (Fig. 2). Otherwise, it appears in vesicles with mesopores uniformly distributed on the surface. The possible reason lies in the difficulty to support a spherical skeleton when the shell of mFePA-HS is too thin [20, 21]. The integrated energy-dispersive X-ray spectroscopy (EDS) analysis elemental mapping images show that C, O, P, and Fe elements are uniformly distributed in the hollow spheres (Fig. 2c), and the atomic composition of C, O, P, and Fe elements is approximately 32.33%, 38.20%, 12.69%, and 16.79%, respectively (Fig. S3). The relative mole ratio of Fe/P in mFePA-HS is calculated to be 0.73/1. The inserted selected-area electron diffraction pattern of mFePA-HS shows no regular lattices, indicating the amorphous frameworks of mFePA-HS (Fig. 2b).

**Fig. 3:**
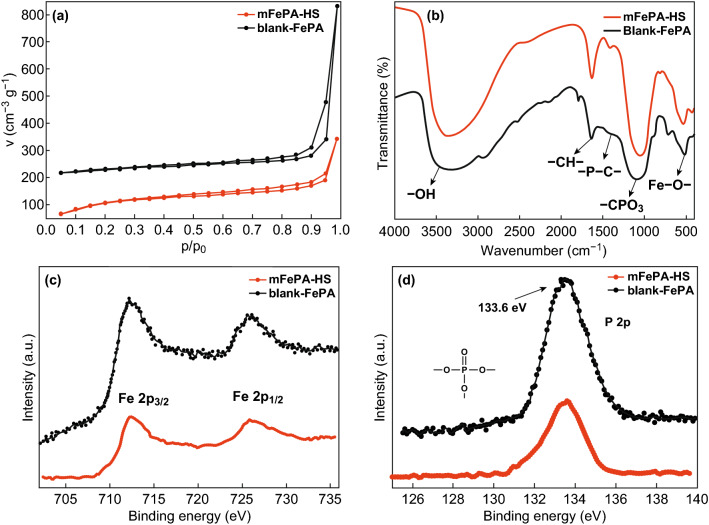
Characterization of mFePA-HS. **a** N_2_ adsorption–desorption isotherms of mFePA-HS and blank-FePA; the curve of blank-FePA has been moved up for 200 cm^−3^ g^−1^. **b** FTIR spectra of the mFePA-HS and blank-FePA.**c** XPS spectra of Fe 2*p*. **d** XPS spectra of P 2*p*

To further survey the pore structure of mFePA-HS, the Brunauer–Emmett–Teller (BET) measurements were taken. The N_2_ adsorption–desorption isotherms of mFePA-HS can be categorized as type IV with a H3 hysteresis loop (Fig. 3a). The appearance of a hysteresis loop (*P*/*P*_o_ of 0.4–0.9) confirms the presence of mesopores caused by the spherical PS-*b*-PEO micelles. Additionally, the BET surface area of the mFePA-HS is 401 m^2^ g^−1^, much higher than that of blank-FePA (108 m^2^ g^−1^) (Table 1). Fourier-transform infrared (FTIR) spectra were also performed to figure out the polymeric framework of materials and evaluate the removal of the templates (Fig. 3b). The peak at 530 cm^−1^ can be ascribed to the stretching vibration of Fe–O; the strong band at 1060 cm^−1^ is associated with the tetrahedral stretching vibration of –CPO_3_ group [22]. Meanwhile, the peaks at 1400 and 1640 cm^−1^ are corresponding to the P–C stretching vibration and the –CH– bending stretching vibration, respectively [13]. In addition, ranging from 500 to 4000 cm^−1^, mFePA-HS exhibits almost the same FTIR signals with blank-FePA, indicating no characteristic peaks of PS-*b*-PEO in the resultant mFePA-HS, as well as –COOH of PFOA. The above results confirm the complete removal of templates as well as the formation of the ferric phytate.

**Table 1:** Porous properties of the mFePA-HS and blank-FePA

Samples	Surface area^a^ (m^2^ g^−1^)	Pore volume (cm^3^ g^−1^)	mesopore size^b^ (nm)
mFePA-HS	401	0.53	12
blank-FePA	108	0.39	–

^a^Surface area was obtained based on the Brunauer–Emmett–Teller (BET) method

^b^The mesopore size was obtained from SEM characterization and averaged at least 50 points

The surface properties of mFePA-HS and blank-FePA were evaluated by XPS. The XPS spectrum of Fe 2*p* shows two main peaks at 725.9 and 712.2 eV, which can be assigned to Fe 2*p*_1/2_ and Fe 2*p*_3/2_ electron bonding energy, respectively. Furthermore, the shake-up peaks at the binding energy of 725.9 eV manifest the + 3 valence state of Fe^3+^ (Fig. 3c) [15]. Figure 3d shows the spectrum of P 2*p*; the intense peak located at 133.6 eV can be assigned to the P 2*p* energy level of pentavalent phosphorus [23]. Powder X-ray (PXRD) diffraction patterns of mFePA-HS and blank-FePA (Fig. S5) give no signatures because of their amorphous structure, consistent with the TEM result (Fig. 2b).

To investigate the component of mFePA-HS, we further conducted a thermogravimetric analysis (TGA) of mFePA-HS with a heating rate of 10 °C min^−1^ under a continuous air flow. The TGA profile shows two stages of weight loss: The first one (20.5 wt%) up to 200 °C is ascribed to the loss of adsorbed water (Fig. S6); and the second one (34.3 wt%) from 200 to 500 °C corresponds to the burning of organics from the framework [13]. Again, it proves that the resultant coordination polymer is composed of organic ligands.

**Fig. 4:**
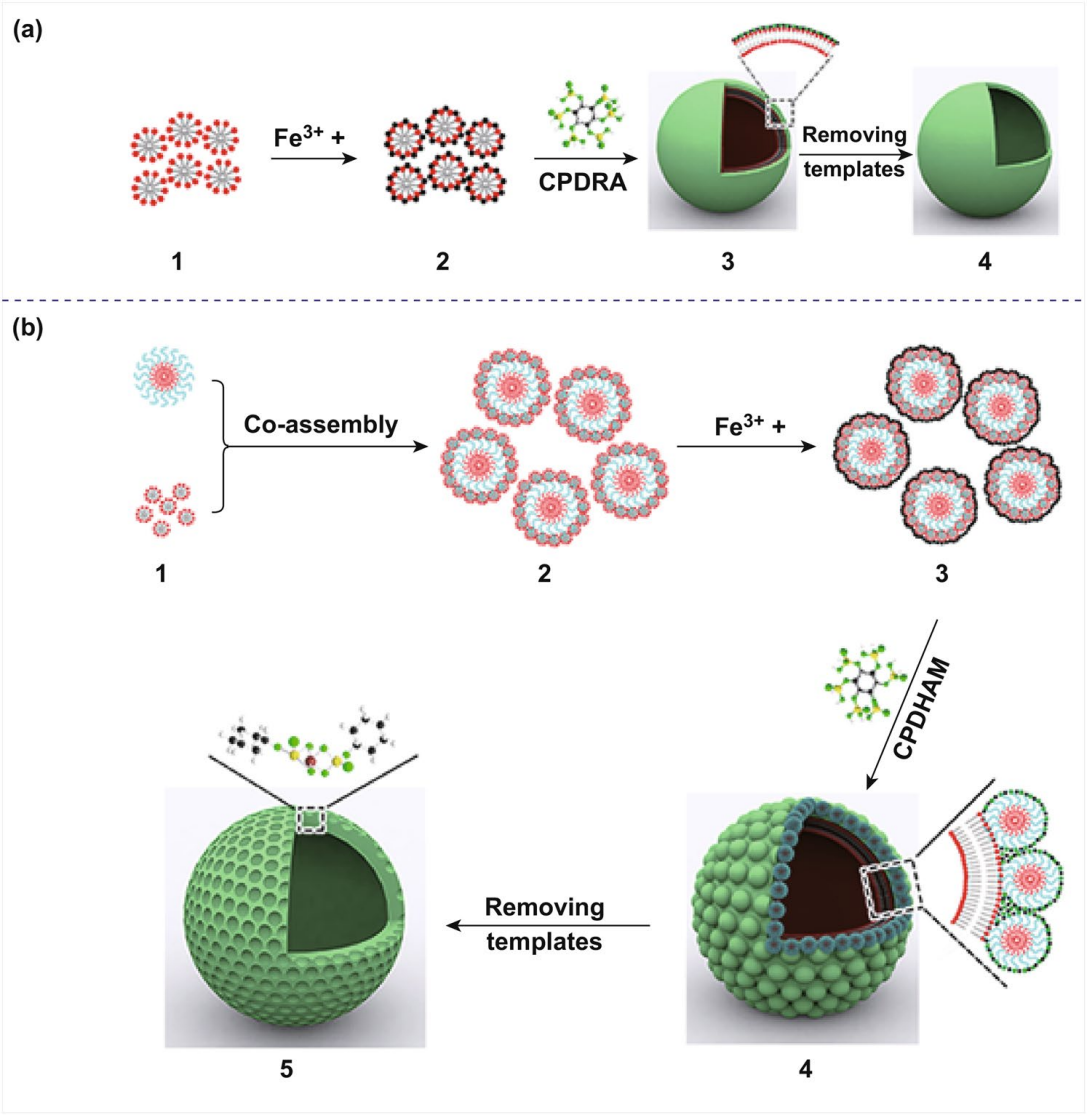
Scheme of the synthesis of hollow FePA nanospheres (**a**) and mFePA-HS (**b**). **a**: (1) The spherical micelles assembled by PFOA. (2) Ferric ions were absorbed on the surface of PFOA micelles due to electrostatic interaction. (3) The formation of pristine ferric phytic acid hollow sphere with the addition of phytic acid by the method of coordination polymerization-driven reassembly (CPDRA) of PFOA micelles. (4) The resultant ferric phytic acid hollow sphere. **b**: (1) The preformed spherical micelles of BCP and PFOA. (2) Hierarchical co-assembly of PFOA and BCP micelles. (3) Ferric ions were absorbed on the surface of PFOA@BCP. (4) The formation of pristine mesoporous ferric phytate hollow sphere with the addition of phytic acid by coordination polymerization-driven hierarchical assembly of micelles (CPDHAM). (5) The obtained mesoporous ferric phytate hollow sphere after the template removal

In order to further clarify the growth mechanism of mFePA-HS, we conducted a series of the controlled experiments. As mentioned above, under the same conditions, only irregular particles of Fe-PA can be obtained without using BCP and PFOA templates (Fig. S7). Similarly, solo BCP also led to irregular particles (Fig. S8). However, hollow FePA nanospheres (PFOA-Fe-phytic acid system) can be generated in the use of PFOA only (Fig. S9). The proposed growth mechanism for the hollow FePA nanospheres is illustrated in Fig. 4a.

As is known, PFOA, a kind of aliphatic fluorosurfactant, can form spherical micelle with a diameter of less than 3 nm once its concentration exceeds critical micelle concentration (CMC) (Fig. 4a-1) [24, 25]. When ferric ions were added into the PFOA solution, they would be absorbed on the surface of the micelle of PFOA due to the electrostatic interaction (Fig. 4a-2). Nonetheless, no obvious assemblies were detected in their solution, as shown by cryogenic transmission electron microscopy (Cryo-TEM), indicating the absence of vesicle in this procedure. Upon the addition of phytic acid, the hollow ferric phytate nanospheres appeared, possibly because that the stronger coordination polymerization interactions between phytic acid and ferric ion induced the rearrangement and reassembly of PFOA into a larger vesicle (Fig. 4a-3) [26].

In the system of mFePA-HS, after the addition of PS-*b*-PEO into the mixed solution of THF/H_2_O, the monodispersed spherical micelles of PS-*b*-PEO with a diameter of 12 nm formed, clearly evidenced by Cryo-TEM (Fig. S10). Subsequently, PFOA ethanol solution was added into BCP solution, the assemblies of PFOA and BCP micelles attract each other to form a hierarchical superstructure by hydrogen bonding (Fig. 4b-2) [27]. This process can be studied by the surface potential change in solution, which decreases from − 6.4 to − 13.1 mV after the addition of PFOA into BCP solution, because of their strong interaction (Figs. S11 and S12). However, Cryo-TEM images do not show changes for the mixed micelles of PFOA and BCP (PFOA@BCP, Fig. S13), possibly due to the lower contrast and smaller size of PFOA micelles. Furthermore, after the positively charged ferric ion was added into the system, the zeta potential of the solution increased up to − 2.61 mV. In this scenario, ferric ion was strongly attracted to the surface of PFOA@BCP assemblies (denoted as Fe@PFOA@BCP, Fig. 4b-3) by the electrostatic interaction between ferric ions and PFOA (Fig. S14). Similarly, Cryo-TEM reveals the spherical morphology of the Fe@PFOA@BCP co-assemblies, without showing visual changes after the addition of ferric ions (Figs. 4b-3 and S15).

**Fig. 5:**
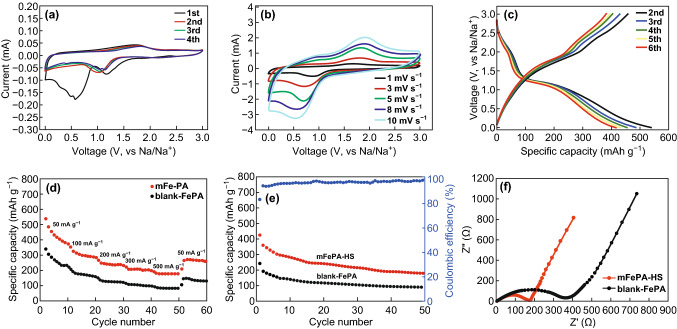
The electrochemical performance of mFePA-HS and blank-FePA. **a** Cyclic voltammetry (CV) curves of mFePA-HS. **b** CV curves of mFePA-HS at different scan rates. **c** The discharge and charge curves after the initial cycle of mFePA-HS. **d** The rate capability of mFePA-HS and blank-FePA. **e** The cycling performance of mFePA-HS and blank-FePA at a current density of 200 mA g^−1^. **f** Nyquist plots of mFePA-HS and blank-FePA at room temperature

However, according to our observation, when the phytic acid molecules were added into the Fe@PFOA@BCP co-assembled solution, the mesoporous FePA hollow nanospheres (mFePA-HS) appeared. In contrast, the PFOA-Fe-phytic acid system also generated the hollow FePA nanospheres, independent of the use of BCP. It means that the intervention of BCP could not interfere with the assembly of PFOA into vesicles. Even at the presence of BCP micelles, the phytic acid molecules can still chelate with ferric ions, which actuate PFOA molecules to rearrange into hollow vesicles (Fig. 4b-4). Moreover, the introduction of BCP endows mFePA-HS with regular mesopores, because BCP micelles could uniformly self-assemble on the surface of the vesicles with the assistance of H-bonding interaction within BCP and PFOA (Fig. 4b-4, b-5). Furthermore, we did a controlled experiment by using octanoic acid as a substitute for PFOA to explore whether the PFOA can be replaced by carboxylic acid surfactants, which possess similar molecular structure. However, as the SEM images show, only hollow ferric phytate nanosphere constructed by irregular particles with no mesoporous can be obtained (Fig. S16). This may be ascribed to the unique hydrophobic fluoroalkyl structure of PFOA [28, 29].The exact mechanism for the growth of mFePA-HS awaits further clarification.

We further evaluated the electrochemical performances of mFePA-HS, as a new type of electrode material for the sodium-ion storage. Figure 5a shows the cyclic voltammetry (CV) curves of mFePA-HS at a low scan rate of 0.1 mV s^−1^. In the first cycle, there is an irreversible peak at ~ 0.56 V, which can be ascribed to the formation of solid electrolyte interphase (SEI) layers, decomposition of electrolyte, and the irreversible sodiation reaction of mFePA-HS [30]. However, in the subsequent cycles, this peak vanishes and the CV curves almost overlap due to the formation of a stable SEI. Furthermore, CV curves of mFePA-HS at different scan rates were obtained, obvious peaks can be observed at cathodic and anodic scans, respectively (Fig. 5b) [31].

Figure 5c depicts the discharge and charge curves after the initial cycle at a current density of 50 mA g^−1^ [32]. The mFePA-HS exhibits a remarkable discharge capacity of 540 mAh g^−1^ in the second cycle, indicating great capability of Na storage [33, 34]. In contrast, the blank-FePA exhibits an inferior electrochemical performance with a lower capacity of 340 mAh g^−1^. The higher capacity of mFePA-HS can be attributed to the unique porous structure, which is beneficial to the sufficient contact with the electrolyte as well as the rapid transport of sodium ions [35, 36]. An obvious plateau can be observed at about 1.1 V in all cycles for the discharging curves. In addition, the discharge/charge profiles almost overlap during the cycles, indicating a good structural stability of the mFePA-HS electrode.

The rate capability of mFePA-HS was evaluated at various current rates from 50 to 500 mA g^−1^. As shown in Fig. 5d, the electrode of mFePA-HS delivers a reversible capacity of 400, 300, 240, 200, and 180 mAh g^−1^ at a current of 50, 100, 200, 300, and 500 mA g^−1^, respectively. Contrastively, the blank-FePA exhibits a much lower rate capability at the same current rates. After changing the current density to 50 mA g^−1^, a reversible capacity of 280 mAh g^−1^ remains, manifesting the good rate capability of mFePA-HS. Figure 5e displays the cycling performance of the electrodes of mFePA-HS and blank-FePA at a current density of 200 mA g^−1^, which were activated at 50 mA g^−1^ in the initial cycle. After 50 cycles, the electrode of mFePA-HS remains at a capacity of 180 mAh g^−1^, far higher than that of blank-FePA (85 mAh g^−1^). In addition, we also evaluated the performance of the mFePA-HS after 500 cycles, the capacity remains 134 mAh g^−1^, which is much better than that of blank-FePA (44 mAh g^−1^) (Fig. S18). Remarkably, although the capacity of mFePA-HS decreases during the cycles, the Coulombic efficiency still approaches about 98%. However, it is worth noting that the cycle stability is also strongly dependent on the compatible electrolyte systems, which should be further investigated.

The effects of the hollow mesoporous structure toward improving the sodium storage performance of the compound were investigated byEIS analysis. Figure 5f presents the Nyquist plots of mFePA-HS and blank-FePA at room temperature, both of which comprise a depressed semicircle in the moderate-frequency area and an inclined line in the low-frequency region [37]. The medium-frequency semicircle normally can be ascribed to the interfacial charge-transfer resistance (*R*_ct_); the obviously smaller diameter of the medium-frequency semicircle of our mFePA-HS electrode reveals its lower *R*_ct_ than that of the blank-FePA, reflecting easier charge-transfer reaction in the current battery system [38]. Furthermore, the low-frequency inclined line is linked to the Warburg impedance (*Z*_w_), which can be interpreted as the diffusion of sodium ion in the host solid-state phase [39].

Finally, SEM images of electrodes before and after cycling were obtained. According to the SEM observation, there are some structural damages in both samples, which may be ascribed to the strong volume change in the electrode. Moreover, comparing the SEM images of blank-FePA electrode before and after cycling, it can be observed that the irregular particles are divided into smaller active particles, indicating the pulverization of the material (Fig. S19) [40]. As known, large volume expansion leads to the pulverization and agglomeration of particles, which could be observed in the SEM images. Furthermore, it brings about the instability of solid electrolyte interface membrane, resulting in the reduction in cyclic stability [41]. In some degree, the problem may be solved by the introduction of volume expansion buffer materials or the construction of microstructures.

The above electrochemical characterization adequately demonstrates that mFePA-HS exhibits superior performance, which is related to the peculiar structure of itself. The open mesopores facilitate the infiltration of electrolyte and significantly shorten the diffusion distance of sodium ions in the solid phase [42]. Additionally, the high surface area would afford more electrochemical reaction interfaces, thus improving the rate capability [35]. Besides, the hollow architecture accommodates volume expansion during the insertion/extraction of sodium [43]. The combination of these features endows the mFePA-HS electrode with the outstanding capacity and reversible electrochemical reaction.

## Conclusions

In summary, we have developed a new coordination polymerization-driven hierarchical assembly of micelle approach for the fabrication of hollow bio-derivatives with ordered mesopores. Driven by the strong coordination between phytic acid and ferric ion, the discrete amphiphilic micelles of PFOA and BCP further rearrange into the hollow vesicles surrounded by the spherical BCP micelles. This procedure accompanies with the confined growth of ferric phytate, enabling the formation of hollow mesoporous ferric phytate. Furthermore, considering other precursors that have similar groups with phytic acid, this method may be appropriate for other acids, such as phosphoric acid, 1-hydroxyethane-1,1-diphosphonic acid, etc.

For the first time, the synchronous control on morphology and mesoscale structure for bio-compounds enriches the material with a large surface area of 401 m^2^ g^−1^, abundant pore volume of 0.53 cm^3^ g^−1^, and regular mesoporous channels of ~ 12 nm. As an unprecedented anode material for sodium-ion batteries, such hollow mesoporous bio-derivative nanospheres exhibit remarkable electrochemical performance. Given the similar coordination ability of the other bio-compounds with metal ions, our study would provide a feasible paradigm for spatially controlled construction of a series of bio-based functional materials.

## Electronic supplementary material

Below is the link to the electronic supplementary material.

Supplementary material 1 (PDF 1540 kb)
