# Supplementary material for: Hollow Bio-derived Polymer Nanospheres with Ordered Mesopores for Sodium-Ion Battery
Source: Nanomicro Lett. 2020 Jan 21;12:31. doi: 10.1007/s40820-020-0370-1 (PMC7770929; doi:10.1007/s40820-020-0370-1)
Supplement: Supplementary file 1 — Supplementary material 1 (PDF 1540 kb) [file 40820_2020_370_MOESM1_ESM.pdf]

Supporting Information for

Hollow Bio-Derived Polymer Nanospheres with Ordered Mesopores for Potential Sodium-Ion Battery

Yan Ai[1], Yuxiu You[2], Facai Wei[1], Xiaolin Jiang[1], Zhuolei Han[1], Jing Cui[1], Hao Luo[1], Yucen Li[1], Zhixin Xu[3], Shunqi Xu[4], Jun Yang[3], Qinye bao[1], Chengbin Jing[1], Jianwei Fu[5], Jiangong Cheng[6], Shaohua Liu[1, 6, *]

[1]State Key Laboratory of Precision Spectroscopy & Department of Materials, School of Physics and Electronic Science, East China Normal University, shanghai 200241, People's Republic of China

[2]Department of Physics and Astronomy, Shanghai Jiao Tong University, Shanghai, 200240 People's Republic of China

[3]School of Chemistry and Chemical Engineering, Shanghai Jiao Tong University, Shanghai 200240, People's Republic of China

[4]Center for Advancing Electronics Dresden (cfaed) &Department of Chemistry and Food Chemistry, Technische Universität Dresden, 01062 Dresden, Germany

[5]School of Materials Science and Engineering, Zhengzhou University, Zhengzhou 450052, People's Republic of China

[6]State Key Lab of Transducer Technology, Shanghai Institute of Microsystem and Information Technology, Chinese Academy of Sciences, Shanghai 200050, People's Republic of China

*Corresponding author. E-mail: shliu@phy.ecnu.edu.cn (Shaohua Liu)

S1 Experimental Section

Chemicals and Materials. BCP of PS_{96}-*b*-PEO_{114} was synthesized in our lab. Phytic acid solution, Iron (III) p-toluenesulfonate and pentadecafluorooctanoic acid were purchased from Maclin, Aladdin and Ark, respectively. Tetrahydrofuran and Ethanol were purchased from Greagent. All chemicals were used without further purification. Deionized water was used for all experiments.

Synthesis of mFePA-HS. Typically, 0.050 g of PS_{96}-*b*-PEO_{114} was dissolved in 1 mL THF and 8 mL H_2O to generate the spherical micelles. After stirring, 0.016 g pentadecafluorooctanoic acid solution (1.1 wt% in ethanol) was added, followed by the addition of 0.040 g iron p-toluenesulfonate (11.8 wt% in water) and the solution turned yellow. Keep stirring for 4 h, the pH of synthetic medium was increased up to 3 by adding 0.140 mL M ammonia. Finally, the reaction vessel was put into ice bath and added 0.010 mL phytic acid solution (70 wt% in water) into it. After continuous

stirring, the color of solution slowly faded, indicating the formation of ferric phytate polymer. The pure mFePA-HS were obtained after removing the BCPs, PFOA and excess ions by repeatedly washing with THF, ethanol, and the resulting product was further dried at 150 °C for 5 h.

Characterization and Measurements. The morphology and structure of the mFePA-HS were investigated by scanning electron microscopy (SEM, S-4800), transmission electron microscopy (JEM-2100F). Infrared spectra were recorded on a FT-IR Spectrometer (Nicolet iS50 FTIR, Thermo). Powder XRD patterns were recorded on a Bruker X-ray diffractometer (Smartlab SE) equipped with Cu-Ka radiation (40 kV, 20 mA) at a rate of 10° min^{-1} over the range 10-80 (2θ). Nitrogen absorption isotherms were measured at 77 K on a Quantachrome 9ASIQMUTV02UT-6. Prior to measurements, all samples were degassed in a vacuum at 120 °C for at least 12 h. Specific surface area was determined by standard Barrett–Emmett–Teller (BET) method in the relative pressure range of 0.05–0.9 P/Po and pore size distribution was analyzed by Density functional theory (DFT). X-ray photoelectron spectroscopy (XPS) measurements were performed in a surface analysis system inducting a sample analysis chamber with the pressure of 3 x 10^{-10} mbar, and the analyzer is Scienta-R3000. The spectra were calibrated by determining to Au $4f_{7/2}$ peak position of the clean Au foil. Thermogravimetric analysis was performed on a TGA/SDTA851e instrument in an air atmosphere. Cryogenic transmission electron microscopy was taken on Tecnai F20 from FEI. Zeta-potential measurements were conducted on Malvern Zetasizer Nano ZS.

Electrochemical Measurements. Electrochemical properties were tested by using a CR2032 coin cell. The working electrodes were prepared by mixing 70 wt% composite materials, 20 wt% carbon black as a conductive agent and 10 wt% polyvinylidene difluoride (PVDF) as binder. After coating the slurry on a Cu foil, the electrodes were dried at 80 °C for 6 h and then transferred to a vacuum oven at 120 °C for 12 h. The loading amount of the electrode was kept at ∼1.5 mg cm^{-2}. The assembly of all coin cells was conducted in an argon-filled glovebox, Sodium metal was used as the counter and reference electrode, 1 M $NaClO_4$ in the mixture of EC/PC (1:1) was used as electrolyte and glass microfiber filters from Whatman were used as separator. Electrochemical tests were conducted by a Land battery system, the voltage window was 0.001−3.0 V. Cyclic voltammetry (CV) tests were carried out by using the three-electrode customized-cell. The CV measurements were performed on a CHI760E electrochemical workstation at a scan rate from o.1–10 mV s^{-1}.

S2 Supplementary Figures

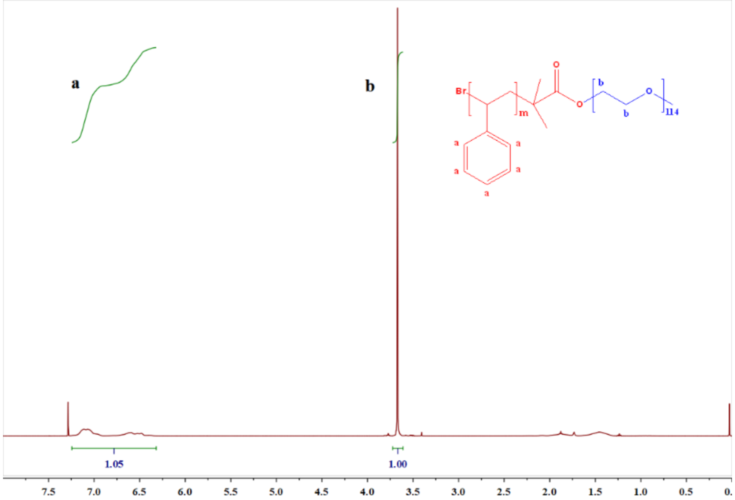

Fig. S1 ^{1}H NMR spectrum of PS$_{96}$-*b*-PEO$_{114}$. The Degree of polymerization (DP) was calculated by the formula: DP= $\frac{Ia/5}{Ib/4}$ $\times 114 = \sim 96$

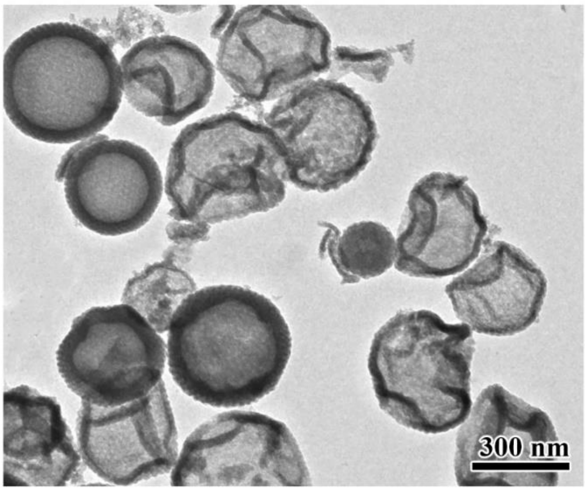

Fig. S2 The TEM image of mFePA-HS

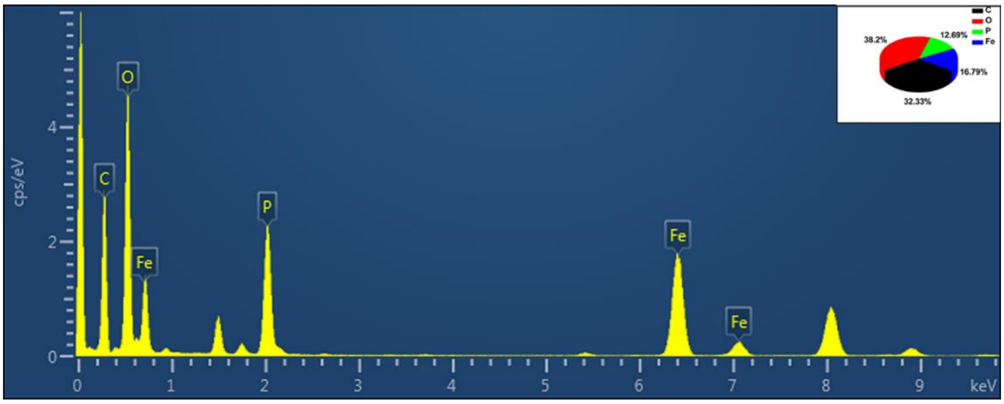

Fig. S3 EDS spectrum of the mFePA-HS

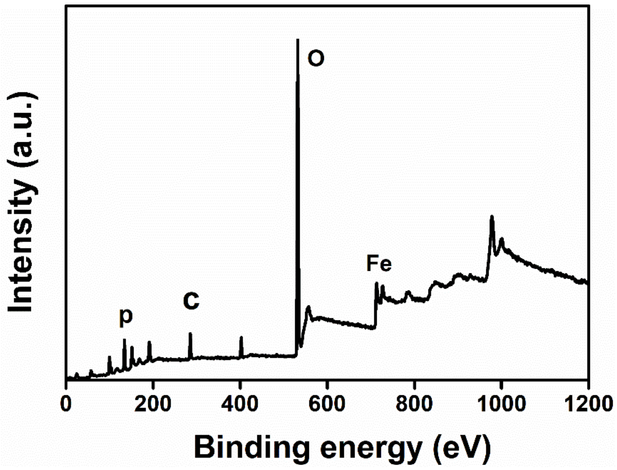

Fig. S4 XPS peak survey spectra of mFePA-HS

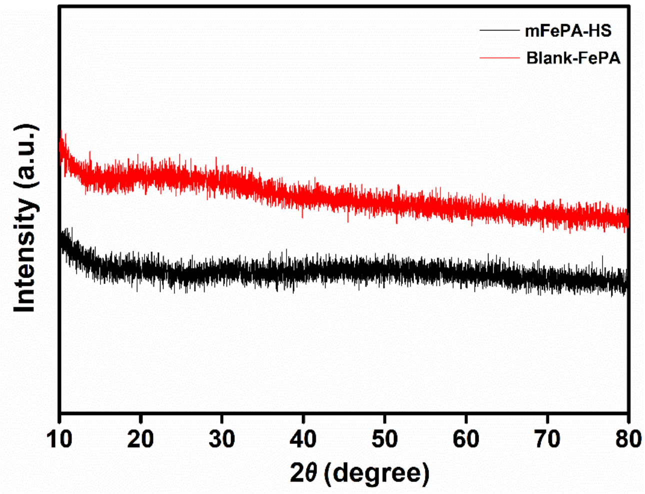

Fig. S5 XRD patterns of the as-made mFePA-HS and the blank-FePA without any templates

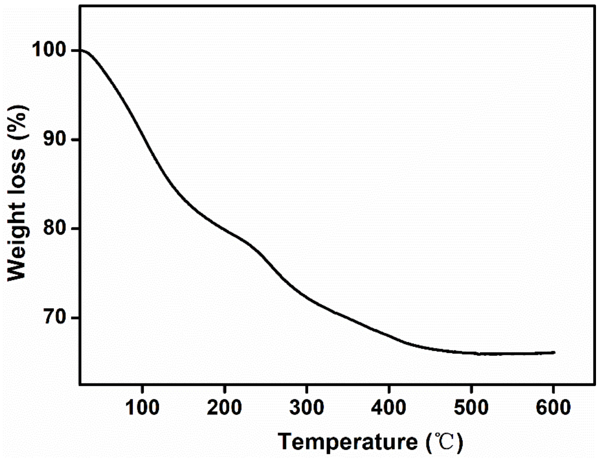

Fig. S6 TGA profile of mFePA-HS

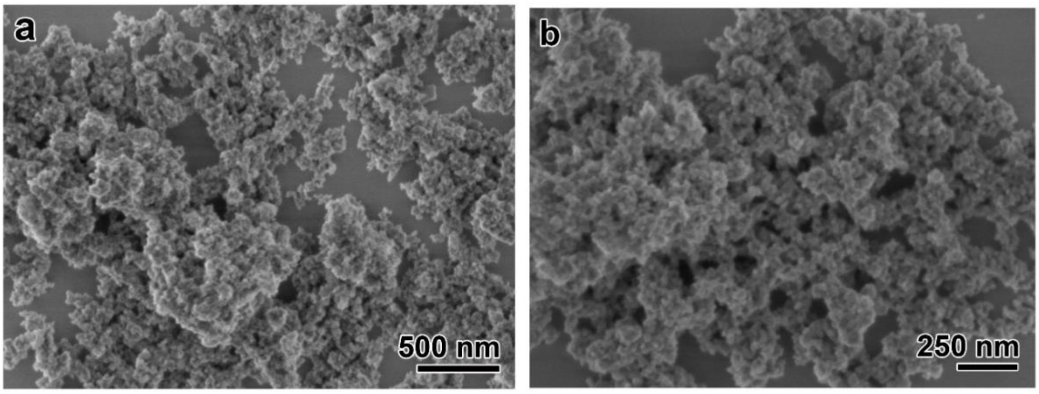

Fig. S7 The blank-FePA irregular particles, which were synthesized without the PFOA and BCP templates. This comparison highlights the critical role of the PFOA and BCP assemblies in the template-directed synthesis of the mesoporous ferric phytic acid hollow sphere

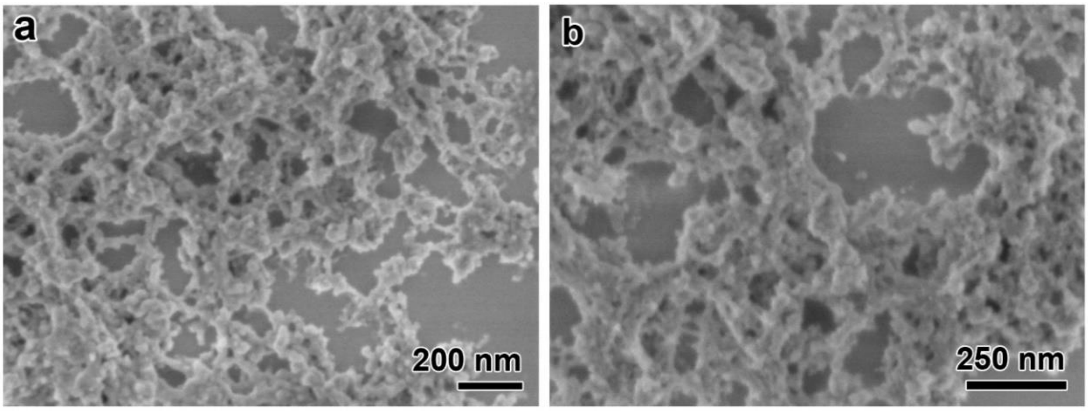

Fig. S8 The blank-FePA irregular particles, which were synthesized with the presence of BCP templates

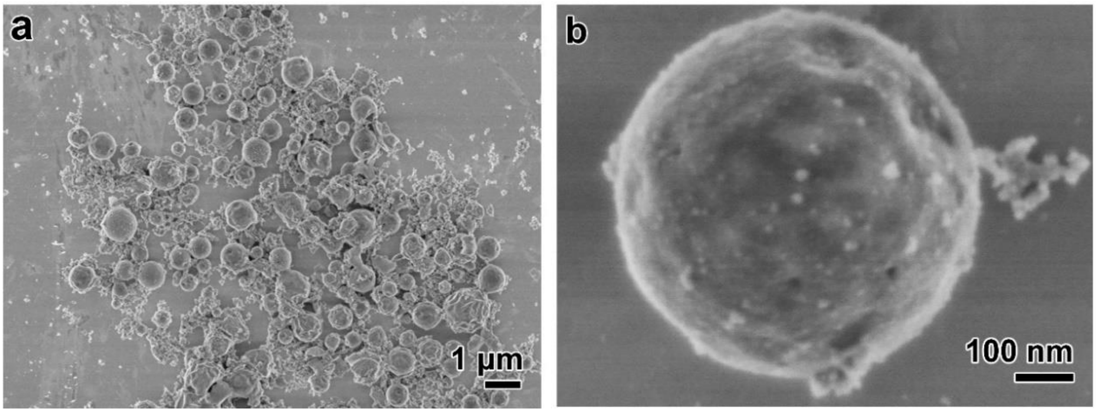

Fig. S9 The blank-FePA hollow sphere, which were synthesized with the presence of PFOA templates

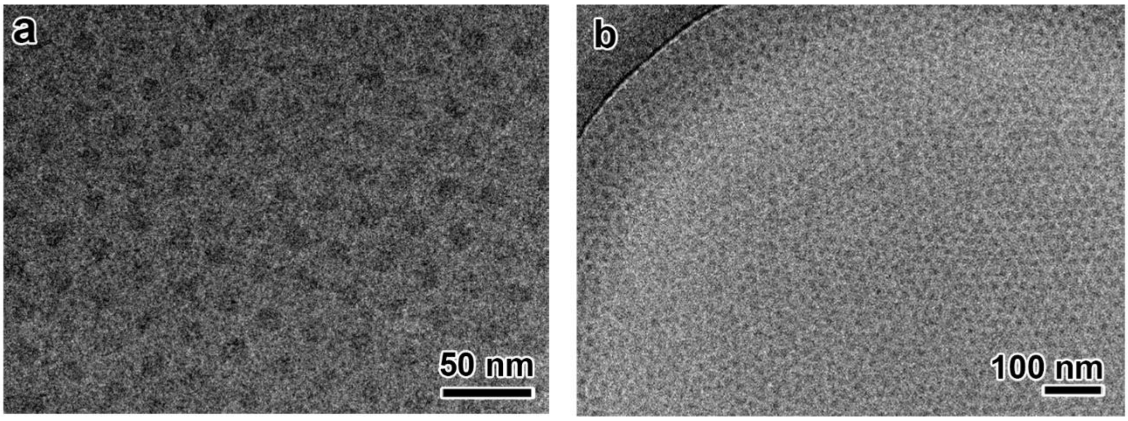

Fig. S10 Cryo-TEM image of PS_{96}-*b*-PEO_{114} BCP micelles

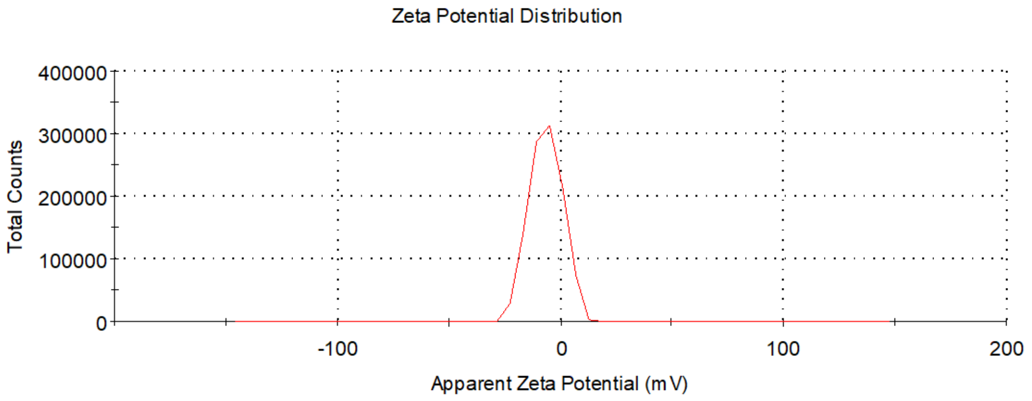

Fig. S11 The zeta potential distribution of solution in the presence of PS-*b*-PEO

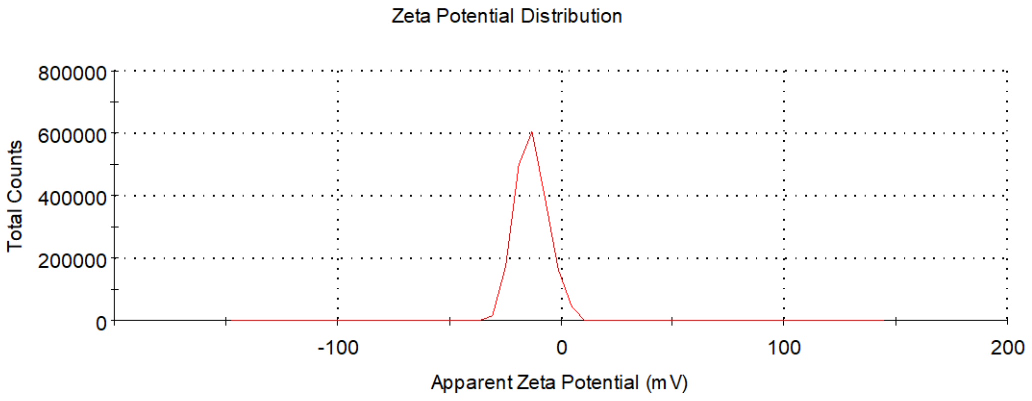

Fig. S12 The zeta potential distribution of solution in the presence of PS-*b*-PEO and PFOA

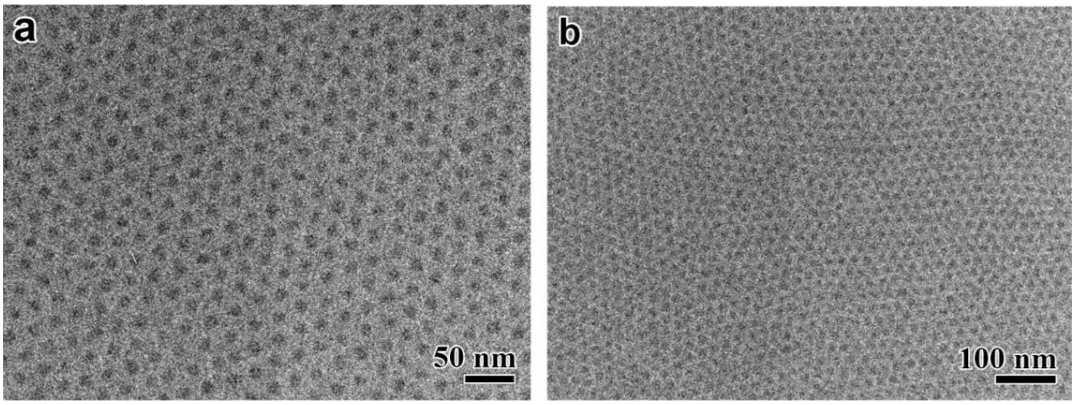

Fig. S13 Cryo-TEM image of PS_{96}-*b*-PEO_{114} BCP micelles and PFOA micelles in the mixed solution of THF, H_2O, and EtOH

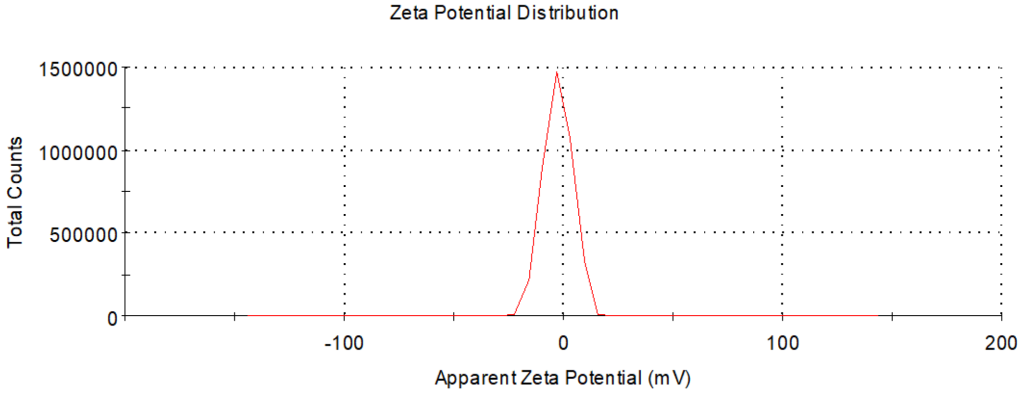

Fig. S14 The zeta potential distribution of solution in the presence of PS-*b*-PEO and PFOA with the addition of Ferric ions

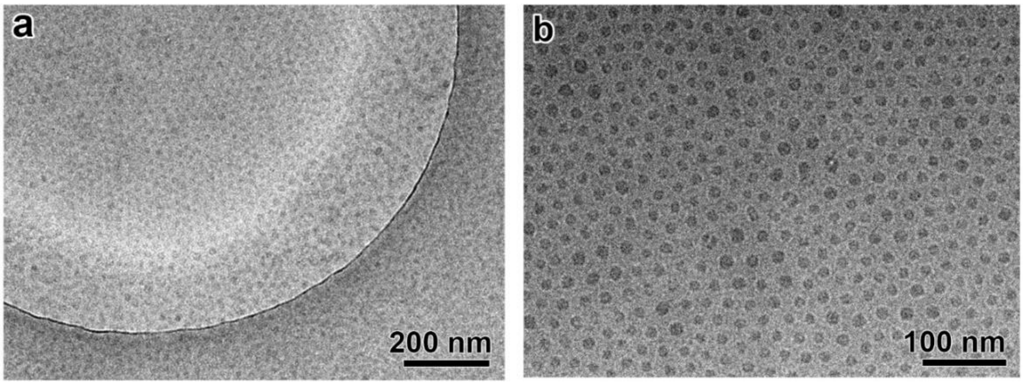

Fig. S15 Cryo-TEM image of PS_{96}-*b*-PEO_{114} BCP micelles and PFOA micelles in the mixed solution of THF, H_2O, and EtOH with the presence of ferric ions

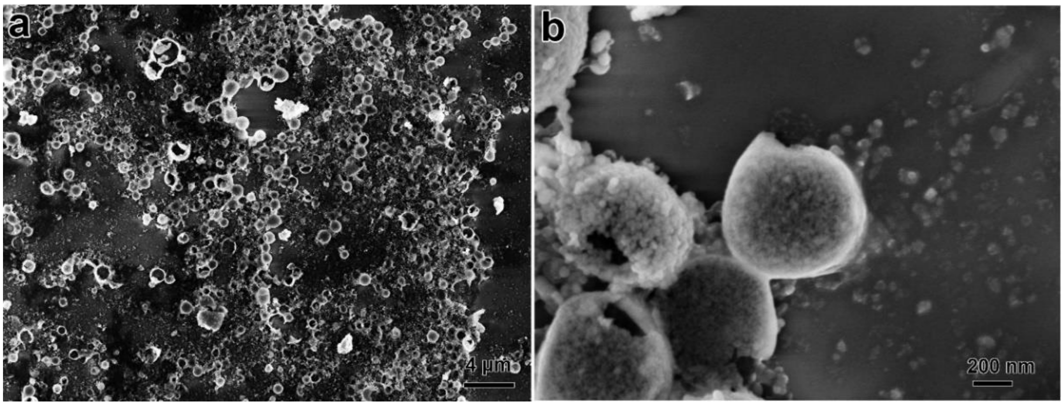

Fig. S16 SEM images of the synthetic ferric phytate at different magnifications by using octanoic acid as a substitute for PFOA

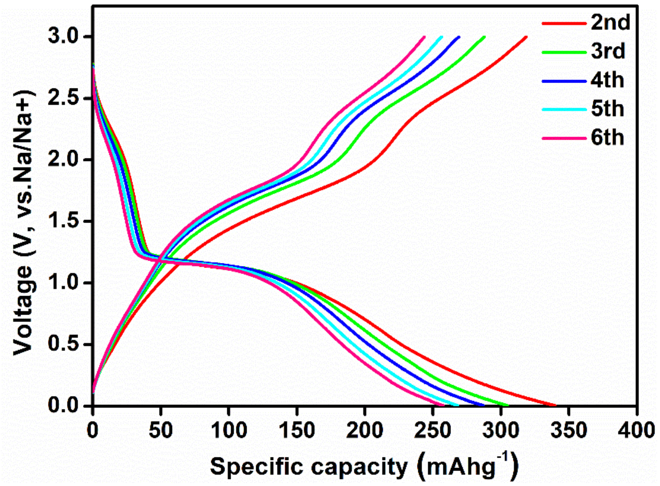

Fig. S17 The discharge and charge curves after the initial cycle of blank-FePA

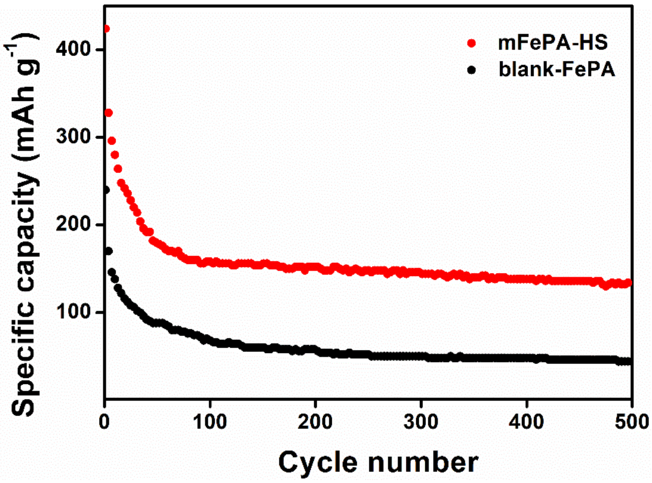

Fig. S18 The cycling performance of mFePA-HS and blank-FePA at a current density of 200 mA g^{-1}

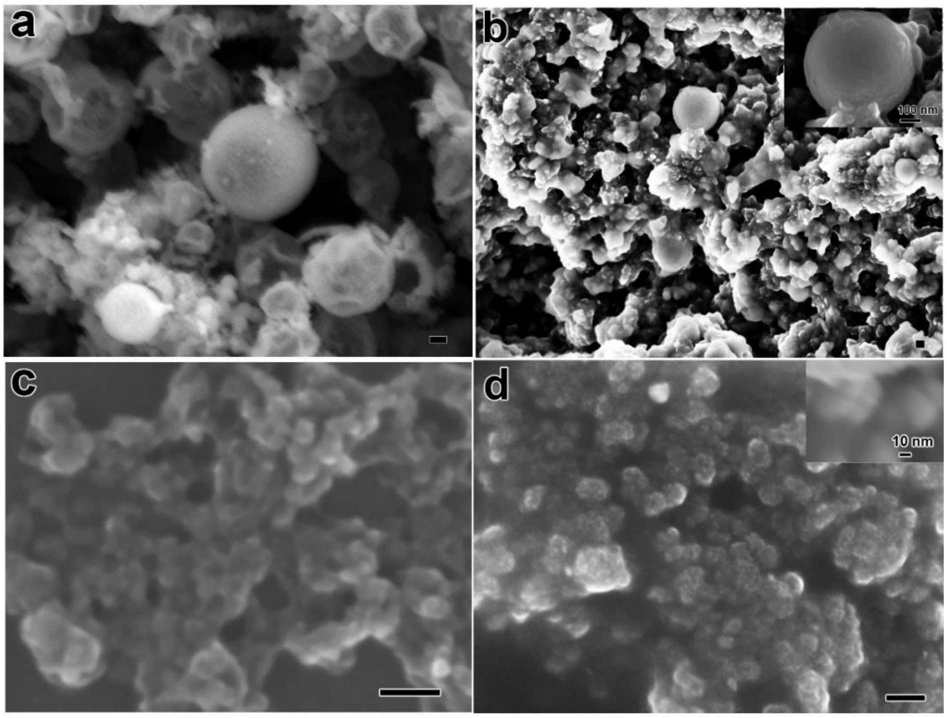

Fig. 19 Various morphologies of electrodes at different states. **a, b** SEM Images of mFePA-HS electrode before and after Cycling. **c, d** SEM Images of blank-FePA electrode before and after Cycling
